# Introduction to the *RSC Advances* Emerging Investigators Series 2023

**DOI:** 10.1039/d4ra90086c

**Published:** 2024-08-27

**Authors:** Fabienne Dumoulin, Shirley Nakagaki

**Affiliations:** a Acibadem Mehmet Ali Aydinlar University Turkey; b Universidade Federal do Paraná Brazil

## Abstract

Dr Fabienne Dumoulin and Professor Shirley Nakagaki are delighted to introduce the *RSC Advances* Emerging Investigators series, which highlights some of the very best work of early career researchers.
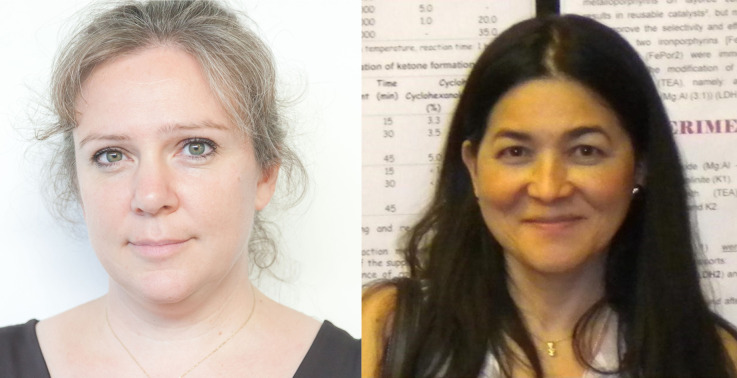

We are proud to present the third edition of the *RSC Advances* Emerging Investigators series (2023 edition). This year we present eight publications selected to support emerging researchers who are already making strides in their respective fields of research, both nationally and internationally. We believe that these researchers will play a crucial role in the scientific landscape, across different areas of knowledge, both in chemistry and in interdisciplinary areas, as evidenced by the articles included here.

We extend our sincere gratitude to all authors for their exceptional contributions, as well as to the editors and referees for their collaboration, which resulted in yet another high-quality edition of the *RSC Advances* Emerging Investigators series.

We kick off our 2023 series with two articles in which researchers have developed analytical tools for the detection of biologically relevant substances.

In the first of these two articles (https://doi.org/10.1039/D3RA04551J) Yangguang Ou and authors from different departments at the University of Vermont, USA, joined forces and expertise to propose a solution for quantifying and understanding the diverse and interesting dynamics that the tryptophan (Trp) amino acid plays in the body. They used fast-scan cyclic voltammetry (FSCV) on carbon fiber microelectrodes (CFMs) for the selective measurement of *in situ* monitoring of Trp in cells, tissues, and *in vivo*. The optimized conditions used led to a sensitive system for detecting Trp, which is selective in the face of over dominant interfering species such as tyrosine (Tyr) amino acid. They used their method for Trp detection in real samples in cultured PC-12 cells and pinealocytes.

In the second article (https://doi.org/10.1039/D3RA06218J), Pavithra Pathirathna *et al.*, researchers from the Department of Chemistry and Chemical Engineering, Florida Institute of Technology, USA, also utilize the FSCV technique and CFM. However, in this work, the researchers present an analytical approach for the preparation and characterization of the double-hole carbon fiber electrode. Although the exact mechanism of the sensor has not yet been explored, the data from this study showed the potential for *in vivo* real-time detection of two analytes simultaneously *via* FSCV, allowing for obtaining missing information about the complex etiology of neurodegenerative diseases.

We also have two articles that showcase researchers’ efforts to prepare and characterize new compounds, particularly investigating their photophysical and photobiological properties to obtain compounds with promising biological activity, for example in drug preparation.

In the first article (https://doi.org/10.1039/D3RA06977J) by Vanessa Nascimento *et al.*, researchers from Brazilian universities (Federal University Fluminense and Federal University of Santa Maria) in various departments (Department of Chemistry, Department of Physics, and Department of Pharmaceutical Technology) joined forces to prepare ten new compounds based on hybrids containing quinones, organochalcogens, and triazoles (1,2,3-triazole naphthoquinones). The new hybrid compounds have pharmaceutical properties, and the materials have a wide range of photophysical properties necessary for the development of advanced devices, such as highly sensitive optical sensors, efficient organic light-emitting diodes (OLEDs), and energy storage and conversion systems. The prepared compounds were characterized by electrochemical analyses, ROS measurements, and antioxidant properties of DPPH, and some of them showed very promising results for future biological applications.

In the second of these two articles (https://doi.org/10.1039/D3RA00823A) a study was conducted by Eufranio N. da Silva Júnior and Bernardo A. Iglesias *et al.* and researchers from two Brazilian universities (Federal University of Santa Maria and Federal University of Minas Gerais) as well as a German university (Julius Maximilians University of Würzburg – JMU). In seeking compounds with fluorescence, the researchers drew inspiration from nature, particularly from lapachol, the synthetic precursor of β-lapachone, a molecule that, through modifications, allows the synthesis of unique heterocyclic compounds with prominent fluorescent properties. In this regard, the authors describe the characterization of a new mono-substituted corrole molecule containing a lapachone unit in one of the *meso* positions. After preparation and characterization, analysis of the photophysical and photobiological characteristics of the new compound revealed that this derivative exhibits some indispensable parameters for a good sensitizer in photodynamic processes, such as photostability, hydrophobic character, and generation of ROS. The authors concluded that lapachone-corroles are promising tetrapyrrolic macrocycles for photobiological applications.

Our 2023 series also contains an interesting study showcasing researchers’ efforts to help address issues around water pollution.

Researchers from Redeemer University and Lead City University, Nigeria, led by Moses O. Alfred and Emmanuel I. Unuabonah, reported for the first time the important monitoring of substances from the dihydroxybenzenes family in water (https://doi.org/10.1039/D3RA04877B). The study provides data on the distribution and toxicity of catechol (CAT) and hydroquinone (HQ) in groundwater and surface waters in three states in southwest Nigeria. Principal Component Analysis (PCA) was used to explain the association between the sources of HQ and CAT in water samples. Assessments of ecological and human health risks were conducted based on the data generated from the analysis of the water samples. Seasonal variation of these substances was observed between rainy and dry seasons, as well as higher average concentrations of CAT compared to HQ (average concentration of CAT in the analyzed samples ranged from 175 to 430 mg L^−1^). It was concluded that the population groups most impacted by these concentrations were children and infants. The results of this study suggest the need for greater control of these dihydroxybenzenes through regular monitoring and removal from drinking water during treatment.

*RSC Advances* also published two very interesting articles that showcase researchers’ efforts in preparing new complex transition metal compounds where the focus is on a deep understanding of structural properties to envision future uses of these compounds.

In the first of these two, researchers from a Brazilian university (Federal University of Rio de Janeiro), led by Giordano Poneti, report the synthesis, structural, spectroscopic, and magnetic investigation of two new cobalt(ii) complexes, introducing the tripodal auxiliary ligand bmimapy ((bis(1-methylimidazol-2-yl)methyl)(2-(pyridyl-2-yl)ethyl)amine) to the community of switchable molecular materials for the preparation of tautomeric valence complexes (https://doi.org/10.1039/D3RA03235C). The authors characterized the prepared compounds using single-crystal X-ray diffraction analysis, magnetic susceptibility and cyclic voltammetry, and the experimental observations were finally justified using DFT analysis, highlighting the ability of the methyl-imidazole pendant arm of the bmimapy ligand to stabilize the high spin Co(ii) redox form and favor the onset of VT phenomenon in cobalt complexes containing bmimapy. They finally concluded that the chemical control of the reduction potential of different metal ions provided by the bmimapy ligand is expected to be a powerful tool to increase the library of tautomeric valence complexes.

The second article reports the efforts of researchers from the University of Windsor, Canada, in better understanding the influence of ligand arrangement in metal complexes and the properties of these complexes (https://doi.org/10.1039/D3RA02797J). The research group, led by Marcus W. Drover, present their recent efforts in understanding a set of ligands containing diboranes, named d*^t^*bbpe, studying their influence on the reactivity of nickel(ii) complexes with nitriles to evaluate the effects of SCS on the resulting product. Additionally, in the work, the authors attempt to relate structural activity to the concentration (*i.e.*, the number) of Lewis SCS acid groups, considering a model reaction of dihydroboration of benzonitrile (PhCN).

Finally, the last article in the 2023 series deals with a theoretical assessment of relevant aspects associated with the drying of colloidal suspensions.

In this work, Henry C. W. Chu *et al.*, researchers from the Department of Chemical Engineering, University of Florida, USA, analyzed, through direct numerical simulations and the development of a macrotransport theory, the role of colloid transport induced by an electrolyte concentration gradient, a mechanism known as diffusiophoresis, in the advective–diffusive transport of an electrolyte-colloid suspension in a unidirectional drying cell under the influence of gravity (https://doi.org/10.1039/D3RA00115F). Drying of a diffusiophoretic colloid suspension attracted by solute causes stronger phase separation and generates a thinner colloidal layer compared to non-diffusiophoretic or solute-repelled colloids. Furthermore, when colloids are strongly repelled by solutes, diffusiophoresis prevents the formation of a colloidal concentration gradient, and therefore gravity has an insignificant effect on the formation of the colloidal layer. The authors also state that their findings are relevant for both terrestrial and space applications.

We hope that the reader finds interesting avenues for future research in each of the areas addressed in the included articles. Additionally, we hope that with this edition, *RSC Advances* fulfils the important role of disseminating quality science carried out by these young researchers, inspiring many others so that we can continue to have new editions of this Emerging Investigators Series.

